# Treatments and outcomes in Chinese patients with serologically active clinically quiescent systemic lupus erythematosus: a retrospective observational study

**DOI:** 10.1186/s13075-021-02641-5

**Published:** 2021-10-29

**Authors:** Hong Huang, Lin Mu, Zhuoli Zhang, Dai Gao, Yanjie Hao, Wei Zhou

**Affiliations:** Department of Rheumatology and Clinical Immunology, Peking University First Hospital, Beijing, 10034 China

**Keywords:** Systemic lupus erythematosus, Disease activity, Outcome, Treatment, Clinically quiescent and serologically active

## Abstract

**Objective:**

To clarify the frequency and outcome of patients with systemic lupus erythematosus (SLE) who achieved the clinical state as serologically active clinically quiescent (SACQ) and to identify factors associated with the flare of disease.

**Methods:**

Clinical data of patients diagnosed as SLE and followed in Peking University First Hospital from 2009 to 2015 were retrospectively reviewed. Six hundred eighty-two patients who were followed up for more than 6 months were analyzed. SACQ was defined as an at least a 6-month period with persistent serologic activity and without clinical activity and daily dose of prednisone or equivalent were less than 7.5 mg. Serologically quiescent clinically quiescent (SQCQ) patients served as control groups. Data including demographics, initial symptoms, duration to SACQ, treatments before and after SACQ, and characteristics of the patients suffered from flare were analyzed.

**Results:**

Among the 682 patients, 170 patients were SACQ (24.9%) and 187 patients were SQCQ. SQCQ patients (38.61 ± 15.08 years old) were older at baseline than SACQ patients (38.61 ± 15.08 years vs. 32.09 ± 14.35 years, *p* < 0.001). Of 170 SACQ patients, 32.9% experienced flare that was significantly higher than 15.5% of SQCQ patients (29/187). Corticosteroids (OR 1.323, 95% CI 1.129 to 1.550; *p* = 0.001) was an independent risk factor for flare, while antimalarials (OR 0.045, 95% CI 0.004 to 0.474; *p* = 0.010) and immunosuppressants (OR 0.332, 95% CI 0.156 to 0.706; *p =* 0.004) were protective factors in SACQ patients; however, only antimalarials was protective factors in SQCQ patients (OR 0.028, 95% CI 0.001 to 0.743; *p =* 0.033).

**Conclusion:**

About one third of SLE patients with SACQ experience flare, significantly more frequent than that of patients with SQCQ. Thus, approach to prevent flare in SACQ patient is required. Maintenance therapy of hydroxychloroquine and immunosuppressant agents may be protective and beneficial treatment strategy in these patients.

**Supplementary Information:**

The online version contains supplementary material available at 10.1186/s13075-021-02641-5.

## Background

Systemic lupus erythematosus (SLE) is a chronic autoimmune disease characterized by multisystem involvements and recurrent flares after the induction of remission. Anti-double-stranded DNA (anti-dsDNA) antibodies are considered as specific markers for SLE present in 60–80% of the patients [1–3]. Anti-dsDNA antibodies have also been recognized as a marker for disease activity, especially for renal injury [4–7]. In addition, these antibodies play a significant role in the exacerbation and flare of the disease [6, 8–11]. Besides anti-dsDNA antibodies, hypocomplementemia has also been identified as a sensitive biomarker of SLE activity and has a predictive effect on the survival and relapse of the disease [11–13].

In recent years, the therapeutic goal for SLE has evolved from a symptom-based to a target-based approach. With the strategy of treat-to-target (T2T) being proposed in 2014, it is a consensus that flare prevention should be a therapeutic goal and that it is a realistic target [14]. And it is also recommended that the maintenance treatment should aim for the lowest glucocorticoid dosage. The Lupus Low Disease Activity State (LLDAS) was proposed in 2015 by the Asia-Pacific Lupus Collaboration as a consensus-based definition of minimally acceptable disease activity lupus patients, with the dosage of prednisone being no more than 7.5 mg/day [15]. And it was reported that in Chinese patients, LLDAS is attainable as an early treatment target for SLE [16]

In 1979, Gladman et al. [17] reported that 14 (8%) of their 180 SLE patients exhibited high levels of serologic markers, anti-dsDNA antibody, and/or hypocomplementemia, without clinical activity, and they described this unique subset of patients as being serologically active clinically quiescent (SACQ) SLE patients.

SACQ patients with SLE appears to account for 6–12% of all patients with SLE, but there is disagreement about whether such patients are indeed clinically stable [17–19]. In the recommendation of the T2T strategies, it was concluded that clinically asymptomatic patients with stable or persistent serological activity should be given closer monitoring instead of treatment escalation [14]. And there is no conclusion as to what kind of treatment should be taken for such patients. In this study, we aimed to investigate the incidence, treatment and prognosis of our SACQ lupus patients.

## Methods

### Study population

This is a retrospective observational study. All consecutive patients recruited in this study were identified from a large, single-center cohort of patients with SLE attending the Rheumatology and Clinical Immunology Department of Peking University First Hospital from January 2007 to December 2015. The patients were followed up for more than 6 months with visits no more than 6 months apart. All patients fulfilled either the 1997 American College of Rheumatology (ACR) Modified Classification Criteria for SLE [20] or the 2012 Systemic Lupus International Collaborating Clinics (SLICC) Classification Criteria [21].

### Definitions

SACQ was defined as an at least a 6-month period with persistent serologic activity and without clinical activity. Each of these patients had a SLE Disease Activity Index 2000 (SLEDAI-2 K) of 2 or 4, from positive anti-dsDNA antibody and/or hypocomplementemia only, at each clinic visit. Serologically quiescent clinically quiescent (SQCQ) patients had at least a 6-month period without serologic or clinical activity (SLEDAI-2 K score = 0). Both SACQ and SQCQ patients could be taking a daily dose of prednisone or equivalent 7.5 mg or less and immunosuppressants with stable or decreasing dosage and/or antimalarials were allowed. The SACQ period was calculated from the first SACQ visit to either the date of known flare or serologic inactivity or to the most recent known SACQ clinic visit. Some patients had more than one SACQ period during follow-up, only the first one was analyzed. Other patients were defined as serologically active clinically active (SACA) in case (1) SLEDAI-2 K score presented from both clinical domain and anti-dsDNA or hypocomplementemia, (2) absent of SLEDAI-2 K score from clinical domain or serological domain less than 6 months or (3) applied more than 7.5 mg prednisone or equivalent per day. Disease flare in clinically quiescent (either SACQ or SQCQ) patients was defined as (1) any increase in the SLEDAI-2 K score accounted by neither hypocomplementemia nor anti-dsDNA, (2) an increase in the prednisone dosage to more than 7.5 mg per day, or (3) judged as flare by physician at a visit during follow-up.

### Data collection

The demographics, laboratory results (anti-dsDNA antibodies, complements), and treatments including corticosteroids, antimalarials, and immunosuppressants were recorded. And corticosteroid doses were converted to milligrams (mg) of prednisone equivalent. All immunosuppressive medicines applied more than 3 months were recorded, including azathioprine 50–100 mg per day, mycophenolate mofetil 0.75–2.0 g per day, methotrexate 7.5–15 mg per week, leflunomide 10–20 mg per day, cyclophosphamide 0.4–0.6 g every 2 weeks and cyclosporin A 100–200 mg per day. SLE-related organ involvements at initial diagnosis, such as lupus nephritis (LN), neuropsychiatric SLE (NPSLE), hematological involvement(thrombocytopenia and hemolytic anemia), pulmonary hypertension, myocardial involvement, interstitial lung disease (ILD), alveolar hemorrhage, smooth muscle involvement, polyserositis and co-morbidities including antiphospholipid antibody syndrome (APS), thrombotic thrombocytopenic purpura (TTP), or thrombotic microangiopathy (TMA), and Sjogren’s syndrome (SS), were collected from the medical records of all recruited patients. The time of the onset of diseases, time to achieve SACQ/SQCQ, duration of SACQ/SQCQ time, and duration of serological activity or inactivity before SACQ/SQCQ were calculated. And for the flare group, the time to first flare after SACQ period and the onset of SLE, the clinical manifestations and SLEDAI-2K score when relapsed, and their prognosis were also collected.

### Statistical analysis

Continuous variables were presented as mean ± standard deviation (SD) or median (interquartile range) for abnormally distributed continuous variables. Categorical variables were described as numbers (percentages or proportions). Normally distributed continuous variables were compared using the Student *t* test with unequal variances, whereas abnormally distributed continuous variables were compared using Mann-Whitney *U* tests. Comparison of categorical data was performed using chi-square tests.

Flare rate over time was estimated using Kaplan-Meier survival analysis, and comparisons of differences in outcome between groups were analyzed using the log-rank test. Logistic regression models, with odds ratios (ORs) and 95% confidence intervals (CIs), were used to determine the variables associated with flare. Age at diagnosis, genders, positive anti-dsDNA antibodies, hypocomplementemia, duration of serological activity or inactivity before SACQ/SQCQ, and therapy including immunosuppressants, corticosteroids, and antimalarials were included in the multivariable Logistic regression model. Besides, as the definitions of disease flare are same in both SACQ and SQCQ groups, we also put the two groups together and tried to identify factors associated with recurrence in these patients.

All reported *p* values are 2-sided and only associations with a *p* value less than 0.05 were considered statistically significant. All statistical analyses were performed using SPSS 20.0.

## Results

**Fig. 1:**
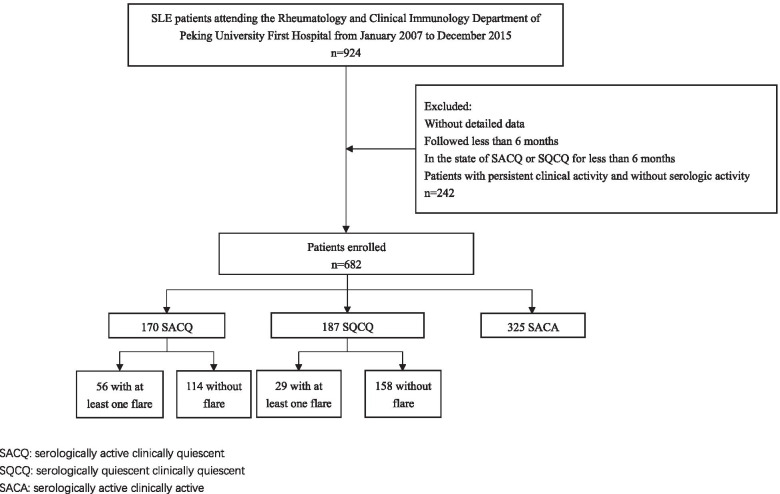
Flow chart of study

A total of 924 SLE patients visited our center between January 2007 and December 2015, and 682 of them were enrolled. We identified 170 patients (24.9%) who fulfilled the SACQ criteria as defined in the “Methods,” and 187 (27.4%) were SQCQ patients. The number of patients who did not achieve clinical remission was 325 (47.7%) (Fig. 1).

The median time from the time of being diagnosed to SACQ occurred was 22.55 (12.70–50.36) months. The patient demographics are summarized in Table 1. We did not find difference in terms of gender between SACQ and SQCQ patients. SQCQ patients were older at study start than did SACQ patients (*p* < 0.001). The SQCQ patients reached the steady state faster and were followed up longer until flare or the last visit than SACQ (*p =* 0.036 and *p =* 0.028, respectively). In the comparison of SACQ and SQCQ, there was no difference in the dose of antimalarials and whether to use immunosuppressants at the start of SACQ or SQCQ (Table 1). The frequency of SLE-related organ involvements and co-morbidities at initial diagnosis in the three cohorts is also outlined in Table 1. There were no between-group differences in the prevalence of SLE-related organ involvements or co-morbidities at the initial diagnosis.

**Table 1:** Patient demographics and symptoms at initial diagnosis

	SACQ (170)	SQCQ (187)	*p*
Age at diagnosis (years)	32.09 ± 14.35	38.61 ± 15.08	< 0.001
Gender (male: female)	21:149	19:168	0.512
Time to get SACQ/SQCQ (months)	22.55 (12.70, 50.36)	18.07 (10.47, 36.53)	0.036
Follow-up time (months)	50.43 (32.93, 82.69)	53.17 (30.40, 80.73)	0.706
The SACQ/SQCQ period (months)	20.30 (10.64, 33.49)	24.00 (12.00, 43.30)	0.028
Dose of antimalarials at the start of SACQ/SQCQ (g)	0.20 (0.20, 0.40)	0.20 (0.20, 0.40)	0.058
Taking immunosuppressants at the start of SACQ/SQCQ (%)	96/170 (56.5%)	99/187 (52.9)	0.504
LN	62 (36.5%)	68 (36.4%)	0.983
NPSLE	8 (4.7%)	11 (5.9%)	0.621
Thrombocytopenia	23/170 (13.5%)	36/187 (19.3%)	0.146
Hemolytic anemia	14/170 (8.2%)	14/187 (7.5%)	0.793
Pulmonary hypertension	4/170 (2.4%)	5/187 (2.7%)	1.000
Myocardial involvement	0/170 (0)	3/187 (1.6%)	0.281
ILD	3/170 (1.8%)	6/187 (3.2%)	0.595
Alveolar hemorrhage	0/170 (0)	0/187 (0)	NA
Smooth muscle involvement	6/170 (3.5%)	5/187 (2.7%)	0.640
Polyserositis	8/170 (4.7%)	8/187 (4.3%)	0.845
APS	7/170 (4.1%)	5/187 (2.7%)	0.450
TTP or TMA	0/170 (0)	0/187 (0)	NA
Secondary SS	19/170 (11.2%)	25/187 (13.4%)	0.529

*SACQ* serologically active clinically quiescent, *SQCQ* serologically quiescent clinically quiescent, *SACA* serologically active clinically active, *NA* not applicable, *LN* lupus nephritis, *NPSLE* neuropsychiatric SLE, *ILD* interstitial lung disease, *APS* antiphospholipid antibody syndrome, *TTP* thrombotic thrombocytopenic purpura, *TMA* thrombotic microangiopathy, *SS* Sjogren syndrome

**Fig. 2:**
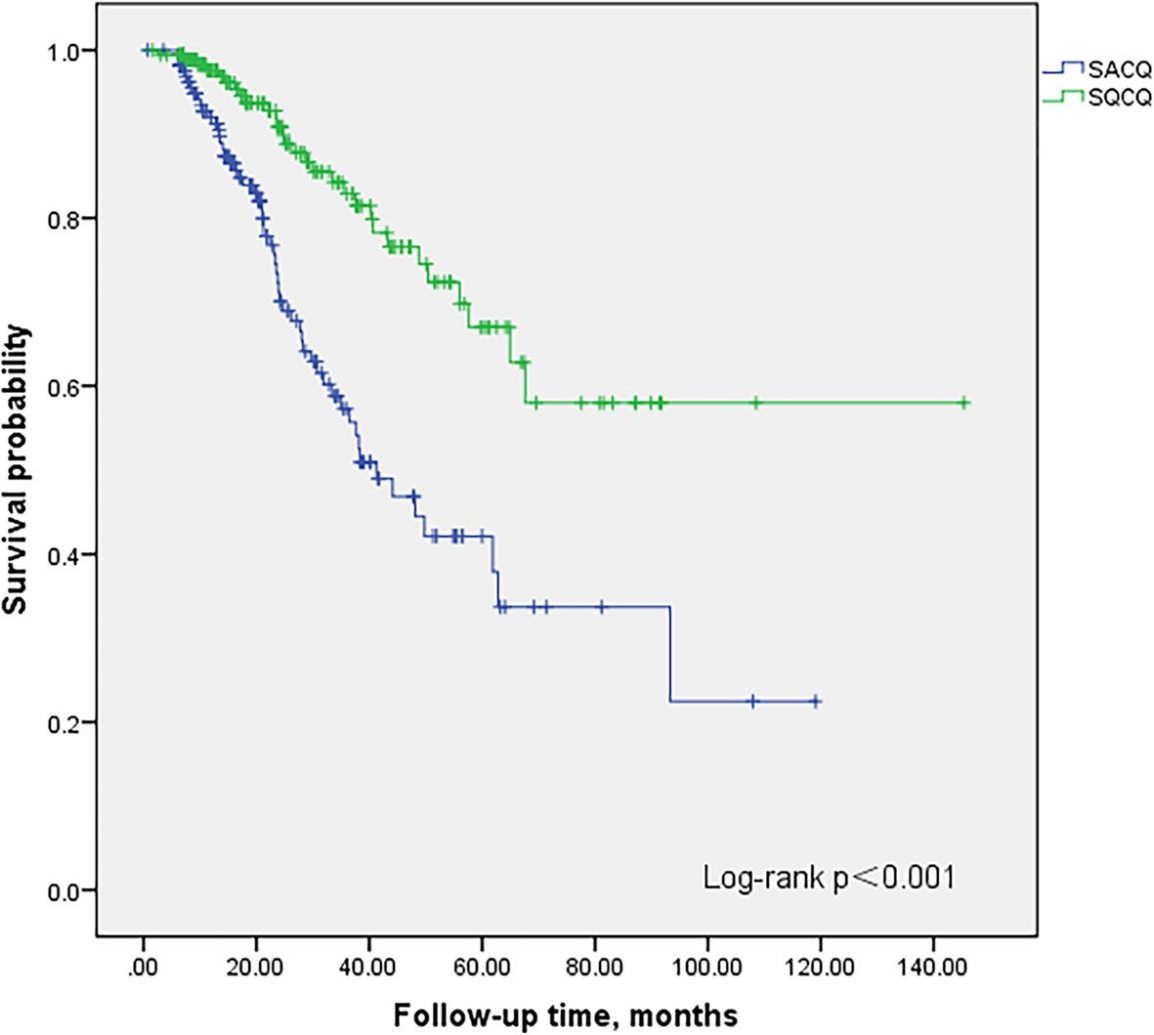
Kaplan-Meier survival curves for SACQ and SQCQ patients

In all patients fulfilled the criteria of SACQ, 114 patients (67.1%) had sustained SACQ state until last visits. There was at least 1 flare in 32.9% (56/170) SACQ patients and 15.5% (29/187) SQCQ patients (*p* < 0.001). Patients with the state of SQCQ were more likely to maintain relapse-free survival than those with the state of SACQ (*p* < 0.001, Fig. 2). Besides, patients in SQCQ group had lower SLEDAI scores than those of SACQ group (*p =* 0.036). The time from they reached the SACQ or SQCQ to recurrence occurred showed a trend of longer time in the SQCQ group than that in the SACQ group, although it was not statistically significant (26.43 months vs. 21.82 months, *p =* 0.079). There was no difference in the treatments of the flare patients between the group of SACQ and SQCQ (Table 2).

**Table 2:** Characteristics of relapsed patients in SACQ and SQCQ group when they relapsed

	SACQ (56)	SQCQ (29)	*p*
Flare	56/170 (32.9%)	29/187 (15.5%)	< 0.001
SLEDAI-2 K	6.0 (4.0, 8.0)	5.0 (2.5, 8.0)	0.036
Recurrence time (month)^a^	21.82 (13.39, 31.59)	26.43 (16.72, 41.97)	0.079
Dose of corticosteroids^b^ (mg)	5.0 (2.81, 7.5)	5.0 (0, 5.0)	0.082
Taking antimalarials	36/56 (64.3%)	17/29 (58.6%)	0.609
Taking immunosuppressants	23/56 (41.1%)	10/29 (34.5%)	0.555

*SLEDAI-2 K* Systemic Lupus Erythematosus Disease Activity Index 2000

^a^The period from the time they reached the SACQ/SQCQ to the time of recurrence

^b^Corticosteroid doses were converted to milligrams (mg) of prednisone equivalent

Neither gender nor age at diagnosis differed between the patients with and without flare in both SACQ group and SQCQ group. And we found no significant difference in the period from the time they reached the SACQ/SQCQ to the time of recurrence or the end of follow-up between the patients who experienced flare and those who did not. Duration of serological activity/inactivity before SACQ/SQCQ was not different between the flare and non-flare group, either. Taking antimalarials when relapsed or at the end of follow-up was significantly different between patients with flare and those without flare in both SACQ (*p =* 0.002) and SQCQ (*p =* 0.001) patients. And the use of immunosuppressants when relapsed or at the end of follow-up followed the same trend in both SACQ (*p =* 0.017) and SQCQ (*p =* 0.041) patients. But the dose of corticosteroids is different between the flare and non-flare group only in SACQ patients (*p* = 0.011). There were no consistent clinical or serologic characteristics that distinguished the recurrent patients from the rest of the SACQ/SQCQ patients (Table 3).

**Table 3:** Comparison of flare and non-flare group in SACQ and SQCQ

	SACQ (170)			SQCQ (187)		
	Flare (56)	Non-flare (114)	*p*	Flare (29)	Non-flare (158)	*p*
Age at diagnosis (years)	30.95 ± 13.88	32.65 ± 14.61	0.469	34.19 ± 14.14	39.42 ± 15.15	0.086
Gender (male to female)	8:48	13:101	0.624	2:27	17:141	0.765
Recurrence time or follow-up time^a^ (months)	21.82 (13.39, 31.59)	19.23 (10.02, 34.32)	0.440	26.43 (16.72, 41.97)	22.92 (11.63, 43.65)	0.361
Duration of serological activity/inactivity before SACQ/SQCQ (months)	13.58 (6.94, 25.19)	15.49 (7.31, 25.87)	0.771	11.10 (5.77, 21.72)	8.88 (3.01, 15.99)	0.115
Time to SACQ/SQCQ^b^ (months)	23.88 (13.03, 50.68)	22.07 (12.16, 50.62)	0.562	21.0 (11.85, 58.65)	17.28 (9.84, 36.53)	0.241
Dose of corticosteroids at the start of SACQ/SQCQ	7.5 (5.0, 7.5)	7.5 (6.25, 7.5)	0.096	7.5 (5.0, 7.5)	7.5 (5.0, 7.5)	0.608
Taking antimalarials at the start of SACQ/SQCQ	40/56 (71.4%)	90/114 (78.9%)	0.277	22/29	132/158	0.319
Taking immunosuppressants at the start of SACQ/SQCQ	29/56 (51.8%)	67/114 (58.8%)	0.414	15/29	84/158	0.886
Dose of corticosteroids^c^ (mg) when relapsed or at the end of follow-up	5.0 (2.81,7.5)	3.75 (1.25, 5.0)	0.011	5.0 (0, 5.0)	3.75 (1.25, 5.0)	0.489
Taking antimalarials when relapsed or at the end of follow-up	36/56 (64.3%)	97/114 (85.1%)	0.002	17/29 (58.6%)	135/158 (85.4%)	0.001
Taking immunosuppressants when relapsed or at the end of follow-up	23/56 (41.1%)	69/114 (60.5%)	0.017	10/29 (34.5%)	87/158 (55.1%)	0.041
Anti-dsDNA (+)	41/56 (73.2%)	96/114 (84.2%)	0.101	—	—	—
Hypocomplementemia	26/56 (46.4%)	36/114 (31.6%)	0.064	—	—	—
Symptoms at initial diagnosis.						
LN	16/56 (28.6%)	46/114 (40.4%)	0.175	10/29 (34.5%)	58/158 (36.7%)	0.819
NPSLE	2/56 (3.6%)	6/114 (5.3%)	1.000	1/29 (3.4%)	10/158 (6.3%)	0.860
Thrombocytopenia	9/56 (16.1%)	14/114 (12.3%)	0.485	6/29 (20.7%)	30/158 (19.0%)	0.831
Hemolytic anemia	5/56 (8.9%)	9/114 (7.9%)	0.776	1/29 (3.4%)	13/158 (8.2%)	0.606
Pulmonary hypertension	3/56 (5.4%)	1/114 (0.9%)	0.105	1/29 (3.4%)	4/158 (2.5%)	1.000
Myocardial involvement	0/56 (0)	0/114 (0)	NA	0/29 (0)	3/158 (1.9%)	1.000
ILD	2/56 (3.6%)	1/114 (0.9%)	0.253	0/29 (0)	6/158 (3.8%)	0.622
Alveolar hemorrhage	0/56 (0)	0/114 (0)	NA	0/29 (0)	0/29 (0)	NA
Smooth muscle involvement	0/56 (0)	6/114 (5.3%)	0.179	0/29 (0)	5/158 (3.2%)	0.730
Polyserositis	2/56 (3.6%)	6/114 (5.3%)	1.000	1/29 (3.4%)	7/158 (4.4%)	1.000
APS	2/56 (3.6%)	5/114 (4.4%)	1.000	1/29 (3.4%)	4/158 (2.5%)	1.000
TTP or TMA	0/56 (0)	0/114 (0)	NA	0/29 (0)	0/29 (0)	NA
Secondary SS	5/56 (8.9%)	14/114 (12.3%)	0.611	3/29 (10.3%)	22/158 (13.9%)	0.823

^a^The period from the time they reached the SACQ/SQCQ to the time of recurrence (flare group) or at the end of follow-up (non-flare group)

^b^The period from the onset of SLE to the time they reached SACQ

^c^Corticosteroid doses were converted to milligrams (mg) of prednisone equivalent

Logistic regression models showed differences in the therapy when the patients relapsed (flare group) or at the end of followed-up (non-flare group) between groups. In the SACQ group, dose of corticosteroids (OR 1.323, 95% CI 1.129 to 1.550; *p* = 0.001) was an independent risk factor for flare, while antimalarials (OR 0.045, 95% CI 0.004 to 0.474; *p* = 0.010) and immunosuppressants (OR 0.332, 95% CI 0.156 to 0.706; *p* = 0.004) were protective factors (Table 4). But in the SQCQ group, we found only antimalarials (OR 0.028, 95% CI 0.001 to 0.743; *p* = 0.033) as protective factors (Table 5). But there was no significant difference in the use of different types of immunosuppressants between the two groups (Table 6).

**Table 4:** Multivariable logistic regressive analysis: risk factors for flare in SACQ

	*β*	*p*	OR (95% CI)
Dose of corticosteroids^a^ (mg) when relapsed or at the end of follow-up	0.280	0.001	1.323 (1.129, 1.550)
Taking antimalarials when relapsed or at the end of follow-up	− 3.105	0.010	0.045 (0.004, 0.474)
Taking immunosuppressants when relapsed or at the end of follow-up	− 1.102	0.004	0.332 (0.156, 0.706)

Variables included in the model: dose of corticosteroids (mg) when relapsed or at the end of follow-up, taking antimalarials when relapsed or at the end of follow-up, taking immunosuppressants when relapsed or at the end of follow-up, duration of serological activity before SACQ (months), anti-dsDNA (+), and/or hypocomplementemia

^a^Corticosteroid doses were converted to milligrams (mg) of prednisone equivalent

**Table 5:** Multivariable logistic regressive analysis: risk factors for flare in SQCQ

	*β*	*p*	OR (95% CI)
Taking antimalarials when relapsed or at the end of follow-up	− 3.584	0.033	0.028 (0.001, 0.743)

Variables included in the model: age at diagnosis (years), dose of corticosteroids (mg) when relapsed or at the end of follow-up, taking antimalarials when relapsed or at the end of follow-up, taking immunosuppressants when relapsed or at the end of follow-up, duration of serological inactivity before SQCQ (months)

**Table 6:** Types of immunosuppressants of flare and non-flare group in SACQ and SQCQ patients

	SACQ (170)			SQCQ (187)		
	Flare (23/56)	Non-flare (69/114)	*p*	Flare (10/29)	Non-flare (87/158)	*p*
AZA	12/23 (52.5%)	27/69 (39.1%)	0.273	2/10 (20%)	23/87 (26.4%)	0.659
MMF	5/23 (21.7%)	23/69 (33.3%)	0.295	1/10 (10%)	21/87 (24.1%)	0.540
MTX	3/23 (13.0%)	11/69 (15.9%)	1.000	6/10 (60%)	23/87 (26.4%)	0.067
LEF	2/23 (8.7%)	8/69 (11.6%)	1.000	5/10 (50%)	20/87 (23.0%)	0.142
CTX	3/23 (13.0%)	4/69 (5.8%)	0.496	2/10 (20%)	6/87 (6.9%)	0.412
CSA	3/23 (13.0%)	1/69 (1.4%)	0.077	0/10 (0%)	4/87 (4.6%)	1.000

*AZA* azathioprine, *MMF* mycophenolate mofetil, *MTX* methotrexate, *LEF* leflunomide, *CTX* cyclophosphamide, *CsA* cyclosporin A

A similar conclusion was reached when the two groups (SACQ and SQCQ patients) were analyzed together (Table 7). The therapy including corticosteroids, antimalarials, and immunosuppressants were related to flare. And the patients in the state of SACQ was also a risk factor for flare (OR 2.550, 95% CI 1.478 to 4.400; *p* = 0.001).

**Table 7:** Multivariable logistic regressive analysis: risk factors for flare

	*β*	*p*	OR (95% CI)
SACQ patients	0.936	0.001	2.550 (1.478, 4.400)
Dose of corticosteroids^a^ (mg) when relapsed or at the end of follow-up	− 1.422	< 0.001	1.239 (1.102, 1.3914)
Taking antimalarials when relapsed or at the end of follow-up	− 1.039	< 0.001	0.241 (0.132, 0.442)
Taking immunosuppressants when relapsed or at the end of follow-up	− 0.945	< 0.001	0.354 (0.202, 0.619)

In both SACQ and SQCQ patients (85 patients). Variables included in the model: SACQ patients or not, age at diagnosis (years), dose of corticosteroids (mg) when relapsed or at the end of follow-up, taking antimalarials when relapsed or at the end of follow-up, taking immunosuppressants when relapsed or at the end of follow-up

^a^Corticosteroid doses were converted to milligrams (mg) of prednisone equivalent

## Discussion

SLE is a chronic disease characterized by varying degrees of severity with multiple episodes of relapse and remission, and the main purpose of treatments includes induction and maintenance of remission. SACQ is a specific subgroup of lupus patients with clinically stable but serological activity. In our cohort, the average age of SACQ was 32.1 years old and was younger than those of SQCQ. Whether these SACQ patients are truly stable is still controversial. Since the first report in 1979 [17], there have been many studies on the disease, and no consensus has been reached on the treatment strategies for these patients.

Previous studies described the natural history of SACQ patients, with varying rates of disease flare from 59 to 81%, given the different definitions of SACQ [19, 22]. These patients were required to be treated without prednisolone or with the mean dose of 5 mg, which were different from ours. This may explain the different rates of relapse. Our patients were allowed to take antimalarials, immunosuppressants, and corticosteroids, but the daily dose of prednisone or equivalent should be no more than 7.5 mg. Therefore, our SACQ patients received more intensive treatments during the maintenance treatment stage. This may be another factor relates to the lower incidence of relapse in our SACQ patients.

Currently, no factors are reliable to predict the course of SLE to maintain remission or to occur relapse. Previous studies have discussed the relationship between serological results and flare, with incongruent results. Steiman AJ et al. [23] investigated the levels of anti-dsDNA and antichromatin isotypes in SACQ patients, and the results showed that predicting clinical outcomes by serologic changes remains an elusive goal among SACQ patients. Ng KP et al. [22] found that anti-dsDNA failed to delineate SACQ patients who will experience flare in the future, but anti-nucleosome (anti-NCS) antibodies might take a role in identifying patients who are more likely to have an earlier flare and multiple flares, especially if they had high titers. In our study, we found no serologic characteristic that may be a predictor for future flares.

Treatment is the focus of our analysis. The target of treatment of SLE should include the prevention of flares [14]. However, our data showed that the incidence of relapse significantly increased in the SACQ patients comparing SQCQ patients. In the 2019 update of the EULAR recommendations for the management of SLE, it was generated that the medium to long-term aim should be to minimize daily dose to ≤ 7.5 mg/day prednisone equivalent or to discontinue them [24]. Our definition of SACQ allow patients take ≤ 7.5 mg/day prednisone equivalent; thus, the treatment and its relationship to relapse of our patients provide information for the therapeutic choices to prevent relapse in SLE patients who may achieve the current treatment targets.

In a prospective, randomized, double-blind, placebo-controlled trial [13], the findings indicated that short-term, moderate-dose corticosteroid treatment can avert severe flares of SACQ patients; thus, they concluded that consideration should be given to the use of corticosteroids for these patients. However, considering the risk of overtreating patients with corticosteroid, the treatment escalation was not recommended for asymptomatic patients with persistent serological activity [14]. A newly published randomized clinical trial found that maintenance of long term 5 mg prednisone in clinically quiescent SLE patients with stable treatment prevents relapse [25]. In our cohort, we found the use of higher dose of corticosteroids related to flare; our data did not support the protective effects of low-dose corticosteroid for preventing relapse in patients with SACQ. As our patients took low-dose corticosteroid to fulfill the diagnosis of SACQ commonly after a tapering period, the relation of usage of higher dose of corticosteroid to flare of lupus may be due to the persistent activity of disease which may in favor to maintaining higher dose of steroid. The effect of low-dose corticosteroid in SLE patients with SACQ on prevention of relapse is still an unsolved problem. However, our study indicated that antimalarials and immunosuppressants were protective factors for flare. This was similar to the results of Conti F et al. [26], who found that the absence of an immunosuppressant should be considered risk factors for the worsening of disease activity in Italian SACQ patients. Due to the diversity of treatments in the observational study and small sample size, we could not find the difference in the effect of different types of immunosuppressants on the relapse. Although the treatment escalation is not recommended for SACQ patients, according to our data, it is reasonable to apply persistent immunosuppressive medicine in these patients, and this may achieve to maintain the immunosuppressant after induction treatment. The optimal therapeutic choice of immunosuppressive medicines to prevent flare of SACQ patients needs further investigation.

There were some limitations of the study, which include its limited number and retrospective nature. Additionally, the conclusions of our study based on Chinese patients may not be generalized to other ethnic populations.

## Conclusion

In conclusion, the clinician needs to pay attention to SLE patients with SACQ since about one third of them experience flare. Our study showed that more intensive treatment strategies, including antimalarials and immunosuppressants, are beneficial to prevent flare in SACQ patients. These results must be validated in other independent cohorts and studies with larger sample size must be undertaken to determine the optimal therapeutic strategy for SACQ SLE patients.

## Supplementary Information

**Additional file 1.**

## Data Availability

All data generated or analyzed during this study are included in this published article and its supplementary information files.

## Abbreviations

SLE: Systemic lupus erythematosus
anti-dsDNA: Anti-double-stranded DNA
T2T: Treat-to-target
LLDAS: Lupus Low Disease Activity State
SACQ: Serologically active clinically quiescent
ACR: American College of Rheumatology
SLICC: Systemic Lupus International Collaborating Clinics
SLEDAI-2 K: SLE Disease Activity Index 2000
SQCQ: Serologically quiescent clinically quiescent
SACA: Serologically active clinically active
LN: Lupus nephritis
NPSLE: Neuropsychiatric SLE
ILD: Interstitial lung disease
APS: Antiphospholipid antibody syndrome
TTP: Thrombotic thrombocytopenic purpura
TMA: Thrombotic microangiopathy
SS: Sjogren’s syndrome
SD: Standard deviation
ORs: Odds ratios
CIs: Confidence intervals
anti-NCS: Anti-nucleosome
