# Supplementary material for: Treatments and outcomes in Chinese patients with serologically active clinically quiescent systemic lupus erythematosus: a retrospective observational study
Source: Arthritis Res Ther. 2021 Oct 29;23:275. doi: 10.1186/s13075-021-02641-5 (PMC8556984; doi:10.1186/s13075-021-02641-5)
Supplement: Supplementary file 1 — Additional file 1. [file 13075_2021_2641_MOESM1_ESM.pdf]

Informed consent

Dear Sir/Madam,

Greetings!

We are going to carry out a study about treatments and outcomes in Chinese patients with serologically active clinically quiescent systemic lupus erythematosus (SLE), and you met our enrollment criteria. Therefore, in order to help us gain a better understanding of SLE and for a better prognosis of the patients, we invite you to participant in this study, and take your following data for publication:

Gender, age, disease duration, SLE-related organ involvements at initial diagnosis, concomitant diseases, SLEDAI-2K score, laboratory results and treatments.

As a retrospective study, you do not have to take any additional examinations.

The results of this study will be kept strictly confidential. Your written consent is required for the researchers to release any data identified with you as an individual to anyone other than personnel working on the project. The information you provide will have your name removed and only a subject number will identify you during analyses and any written reports of the research.

You are volunteering to participate and can withdraw anytime, for any reason, or for no reason at all. You can ask the researchers questions at any time.

I voluntarily agree to participate in this study, and let the researchers use my medical data for publication. I have read and understand the informed consent and conditions of this project. I have had all my questions answered. I hereby acknowledge the above and give my voluntary consent. If I participate, I may withdraw at any time without penalty. I agree to abide by the rules of this project.

Signature： Date：

Researcher: Date：
